# Optimizing Rhamnolipid Performance by Modulating the Expression of Fatty Acid Synthesis Genes *fabA* and *fabZ* in *Pseudomonas aeruginosa* PAO1

**DOI:** 10.3390/genes16050515

**Published:** 2025-04-28

**Authors:** Junpeng Lu, Zhenhua Chen, Huiming Zhu, Qinghai Tang, Zhili Yang

**Affiliations:** Systems Biology Laboratory, School of Marine Science and Technology, Zhejiang Ocean University, Zhoushan 316022, China; lujunpeng233@163.com (J.L.);

**Keywords:** biosurfactant, rhamnolipids, fatty acyl chain, *Pseudomonas aeruginosa*

## Abstract

Background/Objectives: Rhamnolipids (RLs) are biosurfactants with significant industrial and environmental potential, which physicochemical properties depend greatly on their fatty acyl chain composition. This study investigated the impact of genetically modulating the fatty acid synthesis genes *fabA* and *fabZ* on RL composition and functionality in *Pseudomonas aeruginosa* PAO1. Methods and Results: Using temperature-sensitive mutants and suppressor strains for these essential genes, we successfully engineered RLs with altered fatty acyl chain lengths and saturation levels. LC–MS/MS analyses showed that deletion and overexpression of *fabA* and *fabZ* significantly shifted RL fatty acid profiles. Functional analyses indicated that these structural changes markedly influenced RL emulsification activity and critical micelle concentration (CMC). Conclusions: These findings demonstrate the feasibility of optimizing RL properties through targeted genetic manipulation, offering valuable insights for designing customized biosurfactants for diverse industrial and environmental applications.

## 1. Introduction

Rhamnolipids (RLs) are microbial biosurfactants with promising applications in bioremediation, pharmaceuticals, and industrial formulations due to their high surface activity, emulsification capacity, and biodegradability [1,2]. *P. aeruginosa* is one of the most efficient RL producers, synthesizing both mono-rhamnolipids (mRLs) and di-rhamnolipids (dRLs) [3,4]. The fatty acyl composition of RLs, determined by the bacterial fatty acid synthesis (FAS II) pathway, significantly influences their physicochemical properties, including emulsification potential and surface tension reduction [5,6,7,8]. While previous studies have focused on optimizing the RL yield through metabolic engineering and fermentation strategies [9,10,11], limited research has been conducted on tailoring RL functionality by modifying its fatty acyl composition through genetic manipulation.

The fatty acid synthesis pathway in *P. aeruginosa* is governed by the FAS II system, where *fabA* and *fabZ* encode β-hydroxyacyl-ACP dehydratases that regulate acyl chain elongation and desaturation [12,13]. FabA is a bifunctional enzyme catalyzing both the dehydration of β-hydroxyacyl-ACP and isomerization of trans-2-decenoyl-ACP, essential for unsaturated fatty acid biosynthesis [12]. FabZ, a functionally overlapping enzyme, primarily facilitates saturated fatty acid elongation [13,14]. Both *fabA* and *fabZ* genes are essential for bacterial viability [15,16]. Their inactivation leads to cell death, making direct deletion unfeasible under normal growth conditions.

To circumvent this challenge, we previously developed a three-step strategy to construct temperature-sensitive (ts) mutants of essential genes [17,18,19,20]. This method allowed us to generate *fabA* ts-mutants and subsequently isolate suppressor strains that rescued Δ*fabA* lethality, enabling the functional analysis of *fabA* deletion [18]. A similar approach was applied in this study to construct a *fabZ* ts-mutant and screen for suppressor strains that permitted *fabZ* deletion while maintaining bacterial viability. This suppressor-based strategy provides a unique opportunity to investigate how *fabA* and *fabZ* influence RL biosynthesis and its physicochemical properties.

While previous studies have primarily focused on improving the RL yield or general property characterization, relatively few have explored how a targeted modulation of fatty acid biosynthetic genes may influence RL composition and function. This study aims to elucidate the impact of modulating *fabA* and *fabZ* expression on RL acyl chain composition and its emulsification and surfactant properties. We selected *P. aeruginosa* PAO1 as the model organism based on its well-characterized RL biosynthesis pathway [21], which provides both a clearly defined regulatory network and robust genetic tools for precise metabolic engineering. Building on our laboratory’s prior development of a three-step conditional allele construction strategy specifically optimized for this strain, we generated five mutant variants with differential *fabA*/*fabZ* expression profiles while maintaining *rhlC* deletion to ensure exclusive mono-rhamnolipid (mRL) production [22]. Through LC–MS/MS analysis, emulsification assays, and critical micelle concentration (CMC) measurements, we characterized the structure–function relationship of these engineered RL variants. By integrating metabolic engineering with functional analysis, this research contributes to the advancement of customizable RLs biosurfactants, providing a foundation for optimizing RLs for industrial and environmental applications.

## 2. Materials and Methods

### 2.1. Oligonucleotides, Plasmids, and Bacterial Strains

The information on all oligonucleotides, plasmids, and bacterial strains used in this study is summarized in Table 1. The wild-type *P. aeruginosa* PAO1 strain (BioSciBio, Hangzhou, China) and its derivatives were cultured in LB medium (10 g/L tryptone, 10 g/L NaCl, and 5 g/L yeast extract, pH 7.0) or in minimum mineral (MS) medium containing 2% glycerol as the sole carbon source. The MS medium (per liter) consisted of 0.6 g Na_2_HPO_4_, 0.2 g KH_2_PO_4_, 4.0 g NaNO_3_, 0.3 g MgSO_4_, 0.01 g CaCl_2_, and 0.01 g FeSO_4_. The chemicals used in this study were purchased from Macklin Biochemical Co., Ltd. (Shanghai, China).

### 2.2. Plasmid Construction

The deletion and temperature-sensitive (ts) rescue plasmids used in this study were derived from our previously published work [17,18,19,20]. To generate deletion and rescue plasmids, corresponding gene cassettes were amplified by PCR and inserted into the corresponding deletion plasmids and rescue plasmid using the ClonExpress II One Step Cloning Kit (Vazyme, Nanjing, China). For overexpression plasmids, the *araC*-P_BAD_ promoter fragment [23] and the coding sequences of *fabA* or *fabZ* were cloned into the pBBR1MCS-5 vector [24] using the same cloning kit. All recombinant plasmids were confirmed by DNA sequencing prior to use.

### 2.3. Construction of Plasmid-Based Ts-Mutant fabZ(Ts) and ΔrhlC Strains

The *fabZ* temperature-sensitive (ts) mutant strain was constructed using our previously established three-step allelic exchange method [17,18,19,20]. First, a non-replicative *fabZ* deletion plasmid was introduced into *P. aeruginosa* PAO1 via electroporation, and single-crossover integrants were selected on LB plates containing gentamicin. The integrants were then transformed with a temperature-sensitive (ts) rescue plasmid carrying *fabZ* under its native promoter and selected on tetracycline-containing plates. Subsequently, counterselection on sucrose plates (via the *sacB* gene) facilitated excision of the integrated plasmid, generating the chromosomal deletion mutant (Δ*fabZ*). The resulting *fabZ*(Ts) strain was verified by PCR and assessed for temperature-sensitive growth. For *rhlC* gene deletion, a similar two-step allelic exchange approach was employed [25]. Since *rhlC* is a non-essential gene, transformation with a rescue plasmid was not required. The deletion plasmid was introduced into various *P. aeruginosa* strains (PAO1, *fabA*-OE, *fabZ*-OE, Δ*fabA*-sup, and Δ*fabZ*-sup), and single-crossover integrants were selected on tetracycline-containing plates. The final deletion mutants were obtained by sucrose counter-selection and verified by PCR analysis.

### 2.4. Isolation of Suppressors

To obtain spontaneous suppressor mutants, more than 1.0 × 10⁹ *fabZ*(Ts) cells were spread on LB plates and incubated at a semi-restrictive temperature of 40 °C for two weeks, as previously described [17,18]. The plates were kept in a humidified environment with filtered fresh air to support optimal growth. Emergent suppressor colonies were re-streaked on fresh LB plates and incubated at 42 °C to verify the temperature resistance. The presence of the *fabZ* deletion allele was confirmed by PCR using gene-specific primers.

### 2.5. Spot-Plating Assay

Growth of the *fabZ*(Ts) mutant was assessed using a spot-plating assay. Overnight cultures were adjusted to the same OD₆₀₀ and subjected to 10-fold serial dilutions. Diluted samples were spotted onto LB agar plates containing the indicated stress conditions using a 48-pin replicator (V&P Scientific, San Diego, CA, USA). The plates were incubated at 30 °C (permissive temperature) or 42 °C (restrictive temperature), and growth was recorded after appropriate incubation periods.

### 2.6. Rhamnolipid Extraction and Purification

Cell-free supernatants were acidified to pH 2 with HCl to precipitate rhamnolipids and incubated overnight at room temperature. The precipitate was harvested by centrifugation (5000× *g*, 20 min), followed by triple sequential extraction with 100 mL of a 2:1 (*v*/*v*) chloroform–methanol mixture (equal to the initial fermentation volume). After each extraction, the organic phase was collected and concentrated via rotary evaporation. The combined extracts were oven-dried at 70 °C for 12 h to obtain semisolid rhamnolipids. For functional characterization, purified rhamnolipids were dissolved in distilled water (for surface tension and emulsification assays) or chloroform (for TLC and LC–MS/MS analyses).

### 2.7. Quantification of Rhamnolipids via the Orcinol–Sulfuric Acid Method

The orcinol assay was performed using a previously described method [26]. Briefly, 300 μL of culture supernatant was subjected to diethyl ether extraction (1 mL each) after centrifugation. The combined organic phase was dried under vacuum centrifugation and redissolved in 0.5 mL sterile water. Subsequently, 100 μL aliquots were reacted with 900 μL freshly prepared 0.19% (*w*/*v*) orcinol reagent (dissolved in 53% sulfuric acid) for 30 min at 80 °C in a heating block, followed by equilibration to room temperature (15 min). Absorbance readings at 421 nm were acquired using a spectrophotometer. Parallel analysis of rhamnose standards (50–500 mg/L) enabled the construction of a calibration curve by correlating the OD_421_ values with known concentrations.

### 2.8. Thin-Layer Chromatography (TLC) Analysis

Purified rhamnolipids were first dissolved in methanol and then applied onto silica gel TLC plates. The plates were developed using chloroform/methanol/acetic acid (70:10:1.4, *v*/*v*/*v*). After development, the presence of rhamnolipids was detected through staining with anthrone–sulfuric acid and ninhydrin reagents.

### 2.9. Emulsification Capacity Assay

The emulsification index (E24%) was determined following a previously described method [27], with minor modifications. Briefly, equal volumes of two-fold diluted surfactant solution (adjusted to pH 7) and crude oil were mixed vigorously using a vortex mixer (IKA, Staufen, Germany) at maximum speed for 2 min. The mixture was then left undisturbed at 25 °C for 24 h. The E24% was calculated as the height of the emulsion layer divided by the total height of the liquid column, expressed as a percentage.

### 2.10. Determination of the Critical Micelle Concentration (CMC)

The critical micelle concentration (CMC) is defined as the solute concentration at which further increases no longer result in a significant decrease in surface tension. To determine the CMC, the extracted rhamnolipids were dissolved in distilled water to obtain a stock solution with an initial concentration of 96 mg/L. A series of dilutions was then prepared down to 3 mg/L. The surface tension of RL solutions at varying concentrations was measured using a BZY-102 tensiometer (Shanghai Fangrui Instrument Co., Shanghai, China) based on the du Noüy ring method to determine the critical micelle concentration (CMC), and the results were plotted to generate a curve of surface tension versus surfactant concentration. The CMC was identified as the point at which the curve plateaued.

### 2.11. LC–MS/MS Analysis

Purified rhamnolipids were dissolved in chloroform (0.1 g/mL). Analysis was performed using a Waters UPLC system (Waters Corp., Milford, MA, USA) with an Acquity UPLC BEH C18 column (1.7 μm, 2.1 × 50 mm), coupled to an AB Sciex TripleTOF 5600+ mass spectrometer (Sciex, Framingham, MA, USA). The mobile phases were 0.1% formic acid in water (A) and in acetonitrile (B), with a gradient of 20% to 95% B from 0 to 20 min, held until 35 min, and returned to 20% at 36 min. The injection volume was 2 μL, the flow rate 0.4 mL/min, the column temperature 35 °C, and UV detection at 220 nm.

Mass spectrometry was conducted in the negative ion mode for rhamnolipids (scan range *m*/*z* 100–2000), with a source voltage of −4.5 kV and source temperature of 550 °C. Gas pressures were set to 50 psi for Gas 1 and 2 (Air) and 35 psi for Curtain Gas (N_2_). Mass tolerance was ±5 ppm, with a declustering potential of 100 V. Collision energy (CE) was 10 V for MS and 50 ± 20 V for MS/MS, with IRD and IRW set at 67 and 25, respectively. Data acquisition and processing were performed using Analyst TF 1.6 and PeakView v1.2 (AB Sciex, Framingham, MA, USA).

### 2.12. Statistical Analysis

Data are presented as the mean ± standard error. The statistical significance of differences was assessed using an unpaired, two-tailed Student’s *t*-test. A *p*-value < 0.05 was deemed statistically significant.

### 2.13. Data Availability

All the data presented in this document can be found within the manuscript and accompanying Supplementary Files.

## 3. Results

### 3.1. Construction of rhlC-Deleted Strains to Investigate fabA and fabZ Effects on Mono-Rhamnolipid Fatty Acyl Composition

The *fabA* and *fabZ* genes encode β-hydroxyacyl-ACP dehydratases, which are essential for fatty acid biosynthesis and membrane lipid homeostasis in *P. aeruginosa* [12]. Specifically, β-hydroxyacyl-ACP, a precursor for rhamnolipid fatty acyl chains [7], is generated during the fatty acid elongation cycle, thereby influencing the structure of rhamnolipids. Due to their essential roles, the deletion of *fabA* or *fabZ* is lethal. In our previous study, we identified a suppressor strain that rescues the lethal phenotype of the Δ*fabA* mutant (Δ*fabA*-sup), enabling genetic analysis of *fabA*-deficient strains [18]. Similarly, using the same plasmid-based temperature-sensitive (ts) approach, we constructed a *fabZ* ts-mutant *fabZ*(Ts) and subsequently isolated a Δ*fabZ* suppressor mutant, Δ*fabZ*-sup (Supplementary Figure S1), analogous to Δ*fabA*-sup. Additionally, we generated *fabA*-overexpressing (*fabA*-OE) and *fabZ*-overexpressing (*fabZ*-OE) strains [18] using an arabinose-inducible *araC-P_BAD_* promoter system [23].

**Figure 1:**
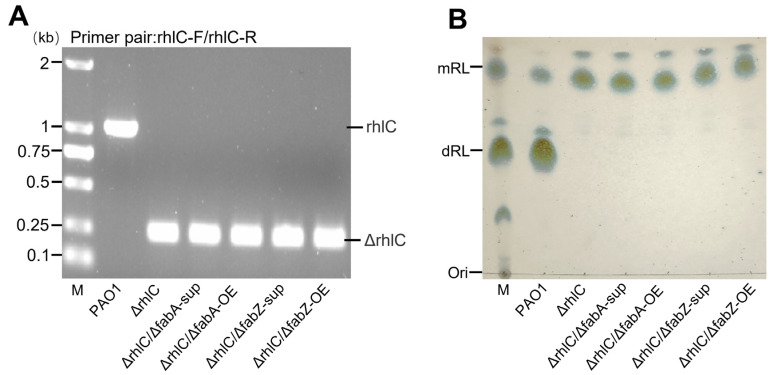
Confirmation of *rhlC* deletion and rhamnolipid composition analysis in mutant strains. (**A**) PCR verification of *rhlC* deletion using the primer pair *rhlC*-F/*rhlC*-R. The wild-type *P. aeruginosa* PAO1 strain shows an intact *rhlC* band (~1 kb), while the Δ*rhlC*-derived mutant strains exhibit a smaller Δ*rhlC* amplicon (~0.2 kb), confirming successful gene deletion. M: DNA marker. (**B**) TLC analysis of rhamnolipid production. The PAO1 produces both mono-rhamnolipids (mRLs) and di-rhamnolipids (dRLs), while Δ*rhlC*-derived mutant strains exclusively produce mRL, confirming the functional loss of *rhlC*. M: rhamnolipid standard marker.

The *rhlC* gene encodes rhamnosyltransferase 2, which converts mono-rhamnolipids (mRLs) into di-rhamnolipids (dRLs) [22]. Therefore, *rhlC* deletion forces the strain to produce only mRLs, providing a suitable model to examine how *fabA* and *fabZ* perturbations affect their fatty acyl chain composition. Using a two-step rapid method for the knockout of dispensable genes in the *P. aeruginosa* method [25] (see Section 2), we deleted *rhlC* in *P*. *aeruginosa* PAO1 wild-type, as well as in the *fabA*-OE, *fabZ*-OE, Δ*fabA*-sup, and Δ*fabZ*-sup backgrounds, generating five mutant strains: Δ*rhlC*, Δ*rhlC*/Δ*fabA*-sup, Δ*rhlC*/*fabA*-OE, Δ*rhlC*/Δ*fabZ*-sup, and Δ*rhlC*/*fabZ*-OE. PCR verification confirmed successful *rhlC* deletion in all strains (Figure 1A), and TLC (thin-layer chromatography) analysis demonstrated that, while wild-type *P. aeruginosa* PAO1 produced both mRLs and dRLs, all mutant strains exclusively synthesized mRLs (Figure 1B).

### 3.2. Effects of fabA and fabZ Deletion and Overexpression on Bacterial Growth and RL Production

To evaluate the impact of *fabA* and *fabZ* deletion and overexpression on bacterial growth and RLs production, we compared the growth curves and rhamnolipid yields of the Δ*rhlC*, Δ*rhlC*/Δ*fabA*-sup, Δ*rhlC*/*fabA*-OE, Δ*rhlC*/Δ*fabZ*-sup, and Δ*rhlC*/*fabZ*-OE strains. All strains were cultured in MS medium supplemented with 2% glycerol, and overexpression of *fabA* and *fabZ* was induced with 0.02% arabinose. The OD_600_ was measured every 12 h, and mRL production was quantified daily.

**Figure 2:**
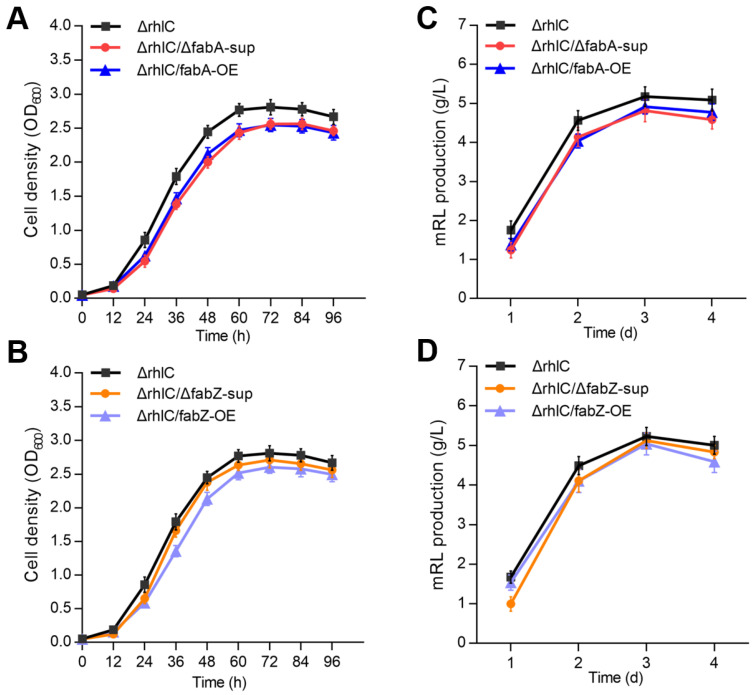
Growth curve analysis and rhamnolipids production of Δ*rhlC*, Δ*rhlC*/Δ*fabA*-sup, Δ*rhlC*/*fabA*-OE, Δ*rhlC*/Δ*fabZ*-sup, and Δ*rhlC*/*fabZ*-OE strains. (**A**,**B**) Growth curves of the Δ*rhlC*, Δ*rhlC*/Δ*fabA*-sup, Δ*rhlC*/*fabA*-OE, Δ*rhlC*/Δ*fabZ*-sup, and Δ*rhlC*/*fabZ*-OE strains, with time (h) on the x-axis and cell density (OD_600_) on the y-axis. (**C**,**D**) Quantification of rhamnolipids by the orcinol method produced by various mutant stains.

Growth curve analysis showed that both *fabA* and *fabZ* deletion, as well as their overexpression, resulted in a slight reduction in growth compared to the control strain Δ*rhlC* (Figure 2A,B and Supplementary Figure S2). Despite these mild differences, all strains reached a stationary phase at approximately 60 h, indicating that *fabA* and *fabZ* mutations do not drastically impair bacterial growth. Similarly, mRL production analysis using an orcinol method [26,28] revealed that *fabA* and *fabZ* suppression or overexpression caused a slight reduction in rhamnolipid yield (Figure 2C,D). However, the differences were relatively minor, suggesting that *fabA* and *fabZ* perturbations do not substantially disrupt rhamnolipid biosynthesis.

### 3.3. Effects of fabA and fabZ Deletion and Overexpression on the Fatty Acyl Composition of mRLs

**Figure 3:**
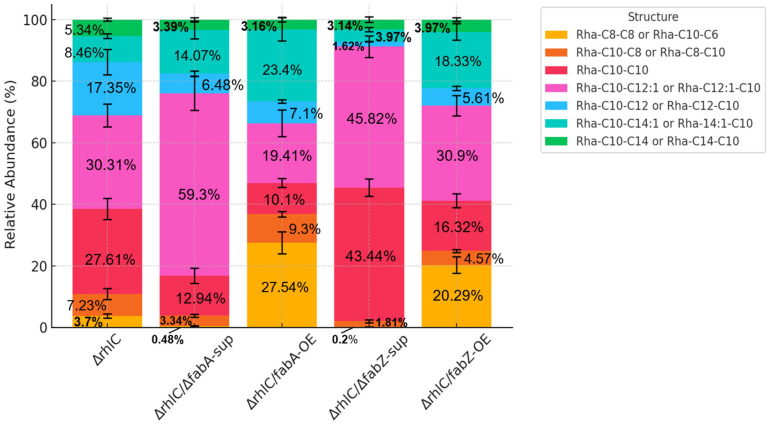
Relative abundance of seven mRL structures in different strains. Stacked bar chart showing the relative abundance of seven major mRL species identified in five *P. aeruginosa* strains: Δ*rhlC*, Δ*rhlC*/Δ*fabA*-sup, Δ*rhlC*/*fabA*-OE, Δ*rhlC*/Δ*fabZ*-sup, and Δ*rhlC*/*fabZ*-OE. The composition of mRL fatty acyl chains was analyzed using LC–MS/MS.

To investigate how *fabA* and *fabZ* deletion or overexpression affects the fatty acyl composition of mRLs, we extracted mRLs from the five strains (Δ*rhlC*, Δ*rhlC*/Δ*fabA*-sup, Δ*rhlC*/*fabA*-OE, Δ*rhlC*/Δ*fabZ*-sup, and Δ*rhlC*/*fabZ*-OE) and analyzed their fatty acyl chain structures using LC–MS/MS. Seven major mRL species were identified based on their negative ion mass spectra (Supplementary Figure S3), and their relative abundances in each strain are summarized in Table 2 and Supplementary Table S1. The results showed that the Δ*rhlC* strain exhibited a balanced distribution of saturated and unsaturated acyl chains, with the most abundant structures being Rha-C10-C10 (27.61%) and Rha-C10-C12:1/Rha-C12:1-C10 (30.31%), followed by Rha-C10-C12/Rha-C12-C10 (17.35%) (Table 2 and Figure 3). However, in Δ*rhlC*/Δ*fabA*-sup, there was a pronounced shift toward longer unsaturated acyl chains, with Rha-C10-C12:1/Rha-C12:1-C10 increasing to 59.30%, while Rha-C10-C10 decreased to 12.94% and shorter acyl chain species (Rha-C8-C8/Rha-C10-C6) were nearly depleted (0.48%) (Table 2 and Figure 3). Conversely, overexpression of *fabA* (Δ*rhlC*/*fabA*-OE) resulted in an increase in Rha-C10-C14:1/Rha-C14:1-C10 (23.40%) and Rha-C8-C8/Rha-C10-C6 (27.54%) compared to their proportions in Δ*rhlC*, while medium-chain fatty acids mRLs such as Rha-C10-C10, Rha-C10-C12:1, and Rha-C12-C10 showed a concurrent decrease (Table 2 and Figure 3).

Similarly, the deletion of *fabZ* also exhibited distinct acyl chain profiles. In Δ*rhlC*/Δ*fabZ*-sup, Rha-C10-C10 increased significantly to 43.44%, accompanied by a notable accumulation of Rha-C10-C12 or Rha-C12-C10 (45.82%) and a sharp decline in longer-chain species such as Rha-C10-C14:1/Rha-14:1-C10 (3.97%) and Rha-C10-C12:1/Rha-12:1-C10 (1.62%) compared to Δ*rhlC* (Table 2 and Figure 3). Overexpression of *fabZ* (Δ*rhlC*/*fabZ*-OE) resulted in a noticeable increase in shorter-chain species of Rha-C8-C8 or Rha-C10-C6 (20.29%) and longer-chain unsaturated species of Rha-C10-C14:1 or Rha-C14:1-C10 (18.33%) compared to Δ*rhlC* (Table 2 and Figure 3). These findings suggest that *fabA* and *fabZ* play regulatory roles in determining the rhamnolipid fatty acid chain length and saturation, likely by modulating the availability of precursor β-hydroxyacyl-ACPs in the fatty acid synthesis pathway.

### 3.4. Emulsification Activity of mRLs with Modified Acyl Chain Composition Due to fabA and fabZ Perturbations

**Figure 4:**
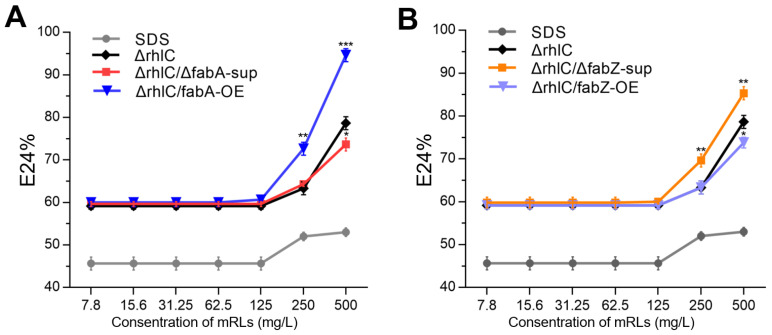
Emulsification activity (E24%) of mRLs produced by different stains. (**A**) Emulsification efficiency of mRLs from the Δ*rhlC*, Δ*rhlC*/Δ*fabA*-sup, and Δ*rhlC*/*fabA*-OE strains. SDS was used as a reference surfactant. Data are the mean ± SD (*n* = 3), Asterisks indicate significant differences (* *p* < 0.05, ** *p* < 0.01, and *** *p* < 0.001; Student’s *t*-test). (**B**) Emulsification efficiency of mRLs from the Δ*rhlC*, Δ*rhlC*/Δ*fabZ*-sup, and Δ*rhlC*/*fabZ*-OE strains.

To assess whether the changes in rhamnolipid acyl chain composition induced by *fabA* and *fabZ* perturbations influence their physicochemical properties, we evaluated the emulsification activity of mRLs produced by the Δ*rhlC*, Δ*rhlC*/Δ*fabA*-sup, Δ*rhlC*/*fabA*-OE, Δ*rhlC*/Δ*fabZ*-sup, and Δ*rhlC*/*fabZ*-OE strains. The emulsification index (E24%) was determined by mixing purified rhamnolipids with crude oil, with SDS (sodium dodecyl sulfate) serving as a reference surfactant (Figure 4A,B).

The results showed that emulsification activity varied significantly among the strains, correlating with their respective acyl chain compositions. At lower concentrations (7.8–125 mg/L), all rhamnolipids exhibited minimal emulsification (E24% ≈ 60%), indicating that higher surfactant concentrations are required for efficient emulsification (Figure 4A,B). However, at concentrations of 250 mg/L and above, clear differences emerged. Among all the strains, Δ*rhlC*/*fabA*-OE (*fabA* overexpression) exhibited the highest emulsification activity, reaching an E24% of nearly 100% at 500 mg/L, significantly higher than the control strain (Δ*rhlC*) (Figure 4A). Conversely, Δ*rhlC*/Δ*fabA*-sup (*fabA* deletion) led to reduced emulsification efficiency. For *fabZ* mutants, Δ*rhlC*/Δ*fabZ*-sup also showed higher emulsification activity than the control (Figure 4B). In contrast, Δ*rhlC*/*fabZ*-OE displayed an emulsification pattern similar to the control Δ*rhlC* strain, with a slight decrease observed at the 500 mg/L RL concentration (Figure 4B). These findings confirm that rhamnolipid emulsification properties can be modulated by altering *fabA* and *fabZ* expressions.

### 3.5. Critical Micelle Concentration (CMC) Analysis of mRLs

**Figure 5:**
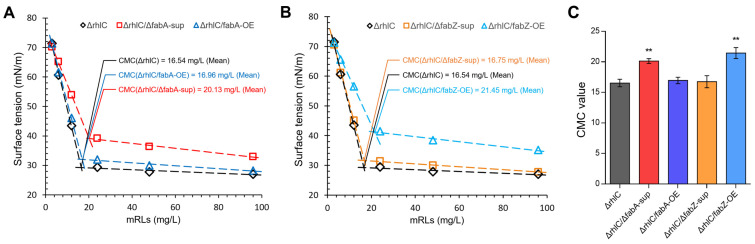
Critical micelle concentration (CMC) of mRLs produced by different stains. (**A**,**B**) The CMC was determined for rhamnolipids extracted from the Δ*rhlC*, Δ*rhlC*/Δ*fabA*-sup, Δ*rhlC*/*fabA*-OE, Δ*rhlC*/Δ*fabZ*-sup, and Δ*rhlC*/*fabZ*-OE strains. CMC values were calculated based on the intersection of two trend lines: the rapid decline in surface tension before micelle formation and the plateau phase after micelle formation. (**C**) Comparison of the CMC values among the five tested strains. Data are the mean ± SD (*n* = 3). ** Indicates a significant difference (*p* < 0.01, Student’s *t*-test).

Surfactants function by reducing surface tension, and their efficiency is often characterized by the critical micelle concentration (CMC), which represents the concentration at which micelles begin to form. A lower CMC value indicates a more efficient surfactant, as micelles are formed at lower concentrations, allowing for more effective surface tension reduction. To evaluate the impact of *fabA* and *fabZ* modifications on rhamnolipid surface activity, we determined the CMC of mRLs produced by the Δ*rhlC*, Δ*rhlC*/Δ*fabA*-sup, Δ*rhlC*/*fabA*-OE, Δ*rhlC*/Δ*fabZ*-sup, and Δ*rhlC*/*fabZ*-OE strains. The results showed that, before reaching the CMC, the surface tension of the rhamnolipid solutions decreased sharply with the increasing concentration. Once a threshold concentration was reached, the reduction in surface tension slowed, eventually stabilizing at a minimum value, beyond which further increases in rhamnolipid concentration had little effect on the surface tension (Figure 5). The intersection of these two trends was used to determine the CMC values of each strain’s rhamnolipids.

The results showed that the CMC values varied considerably among the tested strains, which may correlate with differences in their respective fatty acyl compositions. The control strain (Δ*rhlC*) exhibited a CMC of 16.54 mg/L, reflecting typical rhamnolipid surface activity. *fabA* deletion (Δ*rhlC*/Δ*fabA*-sup) resulted in an elevated CMC of 20.13 mg/L (Figure 5A,C). Conversely, *fabA* overexpression (Δ*rhlC*/*fabA*-OE) reduced the CMC to 16.96 mg/L (Figure 5A,C). Similarly, *fabZ* deletion (Δ*rhlC*/Δ*fabZ*-sup) yielded a resemble CMC (16.75 mg/L) with the Δ*rhlC* strain (Figure 5B,C). In contrast, *fabZ* overexpression (Δ*rhlC*/*fabZ*-OE) resulted in the highest CMC (21.45 mg/L). These results confirm that rhamnolipid surface activity can be affected by altering *fabA* and *fabZ* expression.

## 4. Discussion

With advances in analytical techniques, more than 60 rhamnolipid (RL) congeners have been identified [7], each differing in their glycosyl groups and hydrophobic fatty acyl chains. Variations in the fatty acyl chain length and saturation among these congeners result in distinct physicochemical properties [29,30,31], such as solubility, surface activity, and emulsification efficiency, thus influencing their industrial and environmental applications [8]. Although previous metabolic engineering efforts in RL-producing strains were primarily aimed at increasing RL yields [32,33,34,35], recent findings highlight that precise modulation of the RL fatty acyl composition is equally essential for optimizing their functionality [36,37,38]. In this study, targeted modulation of the fatty acid synthesis genes *fabA* and *fabZ* within the FAS II pathway was shown to influence the fatty acyl chain length and saturation of RLs, offering a novel strategy for customizing RL properties to meet specific application requirements. Critically, this genetic reprogramming strategy maintained host viability, as the modulation of *fabA* and *fabZ* expression exerted only modest effects on bacterial growth and RL output (Figure 2). Both deletion (with suppressors) and mild overexpression caused a slight reduction in the specific growth rate and final RL titer compared to the Δ*rhlC* controls (Figure 2), demonstrating the feasibility of essential gene tuning without compromising RL biosynthesis.

Our results showed that changes in *fabA* and *fabZ* expression do not simply shift RL fatty acyl chains uniformly toward shorter or longer species. Instead, they selectively alter the abundance of specific RL variants. These structural shifts may be mechanistically explained by the altered availability of β-hydroxyacyl-ACP intermediates for RhlA, driven by FabA- and FabZ-mediated fatty acid flux redistribution in the FAS II cycle [5,6,7,8]. For instance, the deletion of *fabA* significantly increased the proportion of medium-chain unsaturated RLs (particularly C10–C12:1) while reducing shorter-chain (C6–C8) and saturated RL species (C10–C10) (Figure 3). Conversely, overexpression of *fabA* increased the proportions of both shorter-chain unsaturated (C8–C10) and longer-chain unsaturated (C14:1) RLs (Figure 3). This likely results from an increased flux through the FabA-mediated dehydration/isomerization step, generating diverse unsaturated intermediates [12,39].

The regulatory effect of *fabZ* exhibited a different pattern. Deletion of *fabZ* enriched the RL populations with medium-length saturated and partially unsaturated fatty acids (notably Rha-C10-C10 and Rha-C10-C12:1/Rha-C12:1-C10), accompanied by reduced longer-chain RLs (Figure 3). Overexpression of *fabZ*, however, produced a bimodal distribution of RL fatty acid lengths (Figure 3), suggesting that excessive FabZ activity disrupts the balance of fatty acid elongation intermediates [13,40], resulting in diverse RL species.

Alterations in the RL structure clearly impacted the emulsification performance. RLs from strains enriched in medium-chain fatty acids (C10–C12), such as Δ*rhlC*/Δ*fabZ*-sup, displayed enhanced emulsification ability (Figure 4B), which may be attributed to the favorable balance between hydrophilic and hydrophobic interactions provided by intermediate-length, partially unsaturated fatty acid chains at the oil–water interface. Conversely, RL mixtures with broader acyl-chain distributions, as in Δ*rhlC*/*fabA*-OE, showed superior emulsification only at higher concentrations, while RLs from Δ*rhlC*/*fabZ*-OE exhibited limited improvements. Critically, while the overall emulsification activity differed among strains, the structural complexity of rhamnolipid mixtures limits the attribution of activity changes to individual congeners alone. Such variability may arise from suboptimal molecular packing at interfaces due to heterogeneity in RL acyl chains [41,42]. However, although both the Δ*rhlC*/Δ*fabA*-sup and Δ*rhlC*/Δ*fabZ*-sup strains produced mRLs enriched in medium-chain fatty acids, only the latter exhibited improved emulsification, suggesting that additional factors such as congener saturation or acyl chain positioning may also contribute to functional differences. Critical micelle concentration (CMC) analysis further supported these observations (Figure 5). RLs from the Δ*rhlC*/Δ*fabZ*-sup and Δ*rhlC*/*fabA*-OE strains maintained relatively low CMC values similar to the control RLs, indicating high surface activity. In contrast, RLs from Δ*rhlC*/*fabZ*-OE and Δ*rhlC*/*fabA*-sup showed a notably higher CMC, reflecting less efficient micelle formation due to diverse fatty acid chain lengths [43,44].

Furthermore, this study highlights the utility of temperature-sensitive alleles and suppressor mutations in functional analyses of essential genes. As the direct deletion of *fabA* or *fabZ* is lethal in *P. aeruginosa* [15], employing suppressor screening methods [17,18] facilitated clear elucidation of their roles in RLs synthesis. Notably, the Δ*rhlC*/Δ*fabZ*-sup and Δ*rhlC*/*fabA*-OE strains exhibited enhanced emulsification activity while maintaining CMC values comparable to the control, highlighting their potential as promising candidates for high-activity RL production. Future studies could extend this approach to other essential fatty acid biosynthesis genes (e.g., *fabB, fabG*, or *fabF*) to further diversify RL structures and optimize their properties. In summary, our study demonstrates that the modulation of *fabA* and *fabZ* significantly influences RL fatty acid composition and functionality, providing both theoretical insights and practical guidelines for engineering RL biosurfactants tailored for specific industrial purposes.

## 5. Conclusions

In this study, targeted genetic modulation of the fatty acid synthesis pathway genes *fabA* and *fabZ* effectively altered the fatty acyl composition of rhamnolipids (RLs) produced by *P. aeruginosa* PAO1. Deletion or overexpression of *fabA* and *fabZ* resulted in distinct and controllable shifts in the RL acyl-chain length and saturation, leading to significant changes in their emulsification performance and surface activity. Additionally, the suppressor-based approach used for analyzing these essential genes provided an effective strategy to dissect their roles in RL biosynthesis. This research provides useful insights into how the genetic modulation of fatty acid synthesis pathways can influence RL structures and function, offering a potential strategy for the development of biosurfactants with improved or application-specific properties.

Nevertheless, this study has certain limitations. First, since *P. aeruginosa* is a human pathogen, large-scale applications necessitate strict containment; future studies should explore transferring these genetic modifications into non-pathogenic hosts. Second, the fatty acid synthesis pathway is regulated by a network of enzymes and factors, and focusing exclusively on *fabA* and *fabZ* may not capture the full spectrum of metabolic control; genes such as *fabB*, *fabG*, or *fabF* may also play significant roles. Addressing these aspects will be essential to further refine and generalize this engineering strategy for broader application.

## Figures and Tables

**Table 1:** Oligonucleotides, plasmids, and strains used in this study.

(A) Oligonucleotides		
Name	Sequence (5′-3′)	Usage
*rhlC*-F	AAGAACGATCATGGACCGGATA	Assay *rhlC* alleles in chr
*rhlC*-R	GAATGCGTTTCGCCGACTAG	Ditto
*fabZ*-F	CACCACGTGCGGACCGATCA	Assay *fabZ* alleles in chr and ts-plasmid
*fabZ*-R	CCTGGCTGCCGTGACCTCAA	Ditto
**(B) Plasmids**		
**Name**	**Relevant Genotype**	**Reference**
pDel	pUC-Gm^r^-sacB	[17,18,19,20]
pTS or pRES	pUC-Tc^r^-ori^ts^	[17,18,19,20]
pOE	pBBRMCS-5-araC-P_BAD_-Gm^r^	[17,18,19,20]
pDel-*fabZ*	*fabZ* Deletion cassette in pDel	This study
pTS-*fabZ*	*fabZ* rescue cassette in pTS	This study
pDel-*rhlC*	*rhlC* Deletion cassette in pDel	This study
pOE-*fabZ*	araC-P_BAD_-*fabZ* in pOE	[18]
pOE-*fabA*	araC-P_BAD_-*fabA* in pOE	[18]
**(C) Strains**		
**Name**	**Relevant Genotype**	**Reference**
PAO1	Wild type *P. aeruginosa*	[17,18,19,20]
Δ*rhlC*	Δ*rhlC* in *P. aeruginosa*	This study
Δ*fabA*-sup	suppressor of Δ*fabA*	[18]
Δ*rhlC*/Δ*fabA*-sup	Δ*rhlC* in Δ*fabA*-sup	This study
*fabA*-OE	pOE-*fabA* in PAO1	[18]
Δ*rhlC*/*fabA*-OE	Δ*rhlC* in *fabA*-OE	This study
Δ*fabZ*-sup	suppressor of Δ*fabZ*	This study
Δ*rhlC*/Δ*fabZ*-sup	Δ*rhlC* in Δ*fabZ*-sup	This study
*fabZ*-OE	pOE-*fabZ* in PAO1	[18]
Δ*rhlC*/*fabZ*-OE	Δ*rhlC* in *fabZ*-OE	This study

**Table 2:** Relative abundance of seven mono-rhamnolipid structures in different strains.

	Formula	M/Z	Δ*rhlC* (% Level, Mean)	Δ*rhlC*/Δ*fabA*-Sup (% Level, Mean)	Δ*rhlC*/*fabA*-OE (% Level, Mean)	Δ*rhlC*/Δ*fabZ*-Sup (% Level, Mean)	Δ*rhlC*/*fabZ*-OE (% Level, Mean)	Structure
1	C_22_H_40_O_9_	448.27	3.70%	0.48%	27.54%	0.20%	20.29%	Rha-C8-C8 or Rha-C10-C6
2	C_24_H_44_O_9_	476.3	7.23%	3.34%	9.30%	1.81%	4.57%	Rha-C10-C8 or Rha-C8-C10
3	C_26_H_48_O_9_	504.33	27.61%	12.94%	10.10%	43.44%	16.32%	Rha-C10-C10
4	C_28_H_50_O_9_	530.35	30.31%	59.30%	19.41%	45.82%	30.90%	Rha-C10-C12:1 or Rha-C12:1-C10
5	C_28_H_52_O_9_	532.36	17.35%	6.48%	7.10%	1.62%	5.61%	Rha-C10-C12 or Rha-C12-C10
6	C_30_H_54_O_9_	557.37	8.46%	14.07%	23.40%	3.97%	18.33%	Rha-C10-C14:1 or Rha-C14:1-C10
7	C_30_H_56_O_9_	559.38	5.34%	3.39%	3.16%	3.14%	3.97%	Rha-C10-C14 or Rha-C14-C10

## Data Availability

The original contributions presented in this study are included in the article/Supplementary Materials. Further inquiries can be directed to the corresponding author.

## Supplementary Materials

The following supporting information can be downloaded at https://www.mdpi.com/article/10.3390/genes16050515/s1: Figure S1: Construction and characterization of the *fabZ* temperature-sensitive (ts) mutant *fabZ*(Ts) and suppressor mutant (Δ*fabZ*-sup) in *P. aeruginosa*; Figure S2: Maximum specific growth rates (μ_max_) of Δ*rhlC*-derived strains; Figure S3: MS2 spectra of various mRL congeners detected; Table S1: Replicate abundance data for mRLs Composition in different strains.

## References

[1] Irfan-Maqsood M., Seddiq-Shams M. (2014). Rhamnolipids: Well-characterized glycolipids with potential broad applicability as biosurfactants. Ind. Biotechnol..

[2] Thakur P., Saini N.K., Thakur V.K., Gupta V.K., Saini R.V., Saini A.K. (2021). Rhamnolipid the Glycolipid Biosurfactant: Emerging trends and promising strategies in the field of biotechnology and biomedicine. Microb. Cell Factories.

[3] Soberón-Chávez G., Lépine F., Déziel E. (2005). Production of rhamnolipids by *Pseudomonas aeruginosa*. Appl. Microbiol. Biotechnol..

[4] Rahman K., Rahman T.J., McClean S., Marchant R., Banat I.M. (2002). Rhamnolipid biosurfactant production by strains *of Pseudomonas aeruginosa* using low-cost raw materials. Biotechnol. Prog..

[5] White S.W., Zheng J., Zhang Y.-M., Rock C.O. (2005). The structural biology of type II fatty acid biosynthesis. Annu. Rev. Biochem..

[6] Zhu K., Rock C.O. (2008). RhlA converts β-hydroxyacyl-acyl carrier protein intermediates in fatty acid synthesis to the β-hydroxydecanoyl-β-hydroxydecanoate component of rhamnolipids in *Pseudomonas aeruginosa*. J. Bacteriol..

[7] Abdel-Mawgoud A.M., Lépine F., Déziel E. (2010). Rhamnolipids: Diversity of structures, microbial origins and roles. Appl. Microbiol. Biotechnol..

[8] Costa S.G., Nitschke M., Lépine F., Déziel E., Contiero J. (2010). Structure, properties and applications of rhamnolipids produced by *Pseudomonas aeruginosa* L2-1 from cassava wastewater. Process Biochem..

[9] Gong Z., He Q., Che C., Liu J., Yang G. (2020). Optimization and scale-up of the production of rhamnolipid by *Pseudomonas aeruginosa* in solid-state fermentation using high-density polyurethane foam as an inert support. Bioprocess Biosyst. Eng..

[10] Blunt W., Blanchard C., Morley K. (2022). Effects of environmental parameters on microbial rhamnolipid biosynthesis and bioreactor strategies for enhanced productivity. Biochem. Eng. J..

[11] Zhao F., Cui Q., Han S., Dong H., Zhang J., Ma F., Zhang Y. (2015). Enhanced rhamnolipid production of *Pseudomonas aeruginosa* SG by increasing copy number of rhlAB genes with modified promoter. RSC Adv..

[12] Heath R.J., Rock C.O. (1996). Roles of the FabA and FabZ β-hydroxyacyl-acyl carrier protein dehydratases in *Escherichia coli* fatty acid biosynthesis. J. Biol. Chem..

[13] Kimber M.S., Martin F., Lu Y., Houston S., Vedadi M., Dharamsi A., Fiebig K.M., Schmid M., Rock C.O. (2004). The structure of (3R)-hydroxyacyl-acyl carrier protein dehydratase (FabZ) from *Pseudomonas aeruginosa*. J. Biol. Chem..

[14] Ruppe S., Mains K., Fox J.M. (2020). A kinetic rationale for functional redundancy in fatty acid biosynthesis. Proc. Natl. Acad. Sci. USA.

[15] Lee S.A., Gallagher L.A., Thongdee M., Staudinger B.J., Lippman S., Singh P.K., Manoil C. (2015). General and condition-specific essential functions of *Pseudomonas aeruginosa*. Proc. Natl. Acad. Sci. USA.

[16] Goodall E.C., Robinson A., Johnston I.G., Jabbari S., Turner K.A., Cunningham A.F., Lund P.A., Cole J.A., Henderson I.R. (2018). The essential genome of *Escherichia coli* K-12. mBio.

[17] Yang Z., Zhang Z., Zhu J., Ma Y., Wang J., Liu J. (2022). Analysis of the plasmid-based ts allele of PA0006 reveals its function in regulation of cell morphology and biosynthesis of core lipopolysaccharide in *Pseudomonas aeruginosa*. Appl. Environ. Microbiol..

[18] Tian L., Yang Z., Wang J., Liu J. (2023). Analysis of the Plasmid-Based ts-Mutant Δ *fabA*/pTS-*fabA* Reveals Its Lethality under Aerobic Growth Conditions That Is Suppressed by Mild Overexpression of desA at a Restrictive Temperature in *Pseudomonas aeruginosa*. Microbiol. Spectr..

[19] Zhang H., Yang Z., Liu J. (2024). Genetic Analysis of the Plasmid-Based Temperature-Lethal Mutant pa1792| lpxH (Ts) in *Pseudomonas aeruginosa*. Genes.

[20] Zhu J., Zhao H., Yang Z. (2024). Genetic Analysis of the ts-Lethal Mutant Δpa0665/pTS-pa0665 Reveals Its Role in Cell Morphology and Oxidative Phosphorylation in *Pseudomonas aeruginosa*. Genes.

[21] Müller M.M., Hörmann B., Syldatk C., Hausmann R. (2010). *Pseudomonas aeruginosa* PAO1 as a model for rhamnolipid production in bioreactor systems. Appl. Microbiol. Biotechnol..

[22] Rahim R., Ochsner U.A., Olvera C., Graninger M., Messner P., Lam J.S., Soberón-Chávez G. (2001). Cloning and functional characterization of the *Pseudomonas aeruginosa rhlC* gene that encodes rhamnosyltransferase 2, an enzyme responsible for di-rhamnolipid biosynthesis. Mol. Microbiol..

[23] Guzman L.-M., Belin D., Carson M.J., Beckwith J. (1995). Tight regulation, modulation, and high-level expression by vectors containing the arabinose PBAD promoter. J. Bacteriol..

[24] Kovach M.E., Elzer P.H., Hill D.S., Robertson G.T., Farris M.A., Roop II R.M., Peterson K.M. (1995). Four new derivatives of the broad-host-range cloning vector pBBR1MCS, carrying different antibiotic-resistance cassettes. Gene.

[25] Huang W., Wilks A. (2017). A rapid seamless method for gene knockout in *Pseudomonas aeruginosa*. BMC Microbiol..

[26] Laabei M., Jamieson W.D., Lewis S.E., Diggle S.P., Jenkins A.T.A. (2014). A new assay for rhamnolipid detection—Important virulence factors of *Pseudomonas aeruginosa*. Appl. Microbiol. Biotechnol..

[27] Cooper D.G., Goldenberg B.G. (1987). Surface-active agents from two Bacillus species. Appl. Environ. Microbiol..

[28] Brückner J. (1955). Estimation of monosaccharides by the orcinol–sulphuric acid reaction. Biochem. J..

[29] Abalos A., Pinazo A., Infante M.R., Casals M., Garcia F., Manresa A. (2001). Physicochemical and antimicrobial properties of new rhamnolipids produced by Pseudomonas a eruginosa AT10 from soybean oil refinery wastes. Langmuir.

[30] Déziel É., Lépine F., Dennie D., Boismenu D., Mamer O.A., Villemur R. (1999). Liquid chromatography/mass spectrometry analysis of mixtures of rhamnolipids produced by *Pseudomonas aeruginosa* strain 57RP grown on mannitol or naphthalene. Biochim. Biophys. Acta (BBA)—Mol. Cell Biol. Lipids.

[31] Monteiro S.A., Sassaki G.L., de Souza L.M., Meira J.A., de Araújo J.M., Mitchell D.A., Ramos L.P., Krieger N. (2007). Molecular and structural characterization of the biosurfactant produced by *Pseudomonas aeruginosa* DAUPE 614. Chem. Phys. Lipids.

[32] Huang C., Li Y., Tian Y., Hao Z., Chen F., Ma Y. (2018). Enhanced rhamnolipid production of *Pseudomonas aeruginosa* DN1 by metabolic engineering under diverse nutritional factors. J. Pet. Environ. Biotechnol..

[33] Sinumvayo J.P., Ishimwe N. (2015). Agriculture and food applications of rhamnolipids and its production by *Pseudomonas aeruginosa*. J. Chem. Eng. Process Technol..

[34] Chong H., Li Q. (2017). Microbial production of rhamnolipids: Opportunities, challenges and strategies. Microb. Cell Factories.

[35] Pang A.-P., Wang Y., Zhang T., Gao F., Shen J.-D., Huang L., Zhou J., Zhang B., Liu Z.-Q., Zheng Y.-G. (2024). Highly efficient production of rhamnolipid in P. putida using a novel sacB-based system and mixed carbon source. Bioresour. Technol..

[36] Sakthipriya N., Doble M., Sangwai J.S. (2015). Biosurfactant from Pseudomonas species with waxes as carbon source–Their production, modeling and properties. J. Ind. Eng. Chem..

[37] Wittgens A., Santiago-Schuebel B., Henkel M., Tiso T., Blank L.M., Hausmann R., Hofmann D., Wilhelm S., Jaeger K.-E., Rosenau F. (2018). Heterologous production of long-chain rhamnolipids from Burkholderia glumae in Pseudomonas putida—A step forward to tailor-made rhamnolipids. Appl. Microbiol. Biotechnol..

[38] Tiso T., Zauter R., Tulke H., Leuchtle B., Li W.-J., Behrens B., Wittgens A., Rosenau F., Hayen H., Blank L.M. (2017). Designer rhamnolipids by reduction of congener diversity: Production and characterization. Microb. Cell Factories.

[39] Xiao X., Yu X., Khosla C. (2013). Metabolic flux between unsaturated and saturated fatty acids is controlled by the FabA: FabB ratio in the fully reconstituted fatty acid biosynthetic pathway of *Escherichia coli*. Biochemistry.

[40] Dodge G.J., Patel A., Jaremko K.L., McCammon J.A., Smith J.L., Burkart M.D. (2019). Structural and dynamical rationale for fatty acid unsaturation in *Escherichia coli*. Proc. Natl. Acad. Sci. USA.

[41] Guzmán E., Ortega F., Rubio R.G. (2024). Exploring the world of rhamnolipids: A critical review of their production, interfacial properties, and potential application. Curr. Opin. Colloid Interface Sci..

[42] Palos Pacheco R., Kegel L.L., Pemberton J.E. (2021). Interfacial and solution aggregation behavior of a series of bioinspired rhamnolipid congeners rha-C14-c x (x= 6, 8, 10, 12, 14). J. Phys. Chem. B.

[43] Mitrinova Z., Tcholakova S., Popova Z., Denkov N., Dasgupta B.R., Ananthapadmanabhan K. (2013). Efficient control of the rheological and surface properties of surfactant solutions containing C8–C18 fatty acids as cosurfactants. Langmuir.

[44] Youssef N.H., Nguyen T., Sabatini D.A., McInerney M.J. (2007). Basis for formulating biosurfactant mixtures to achieve ultra low interfacial tension values against hydrocarbons. J. Ind. Microbiol. Biotechnol..

