# Supplementary material for: Optimizing Rhamnolipid Performance by Modulating the Expression of Fatty Acid Synthesis Genes fabA and fabZ in Pseudomonas aeruginosa PAO1
Source: Genes (Basel). 2025 Apr 28;16(5):515. doi: 10.3390/genes16050515 (PMC12111694; doi:10.3390/genes16050515)
Supplement: Supplementary file 1 [file genes-16-00515-s001.zip › fabAZ_mRL_Supplementary Figure S1-S3 .pdf]

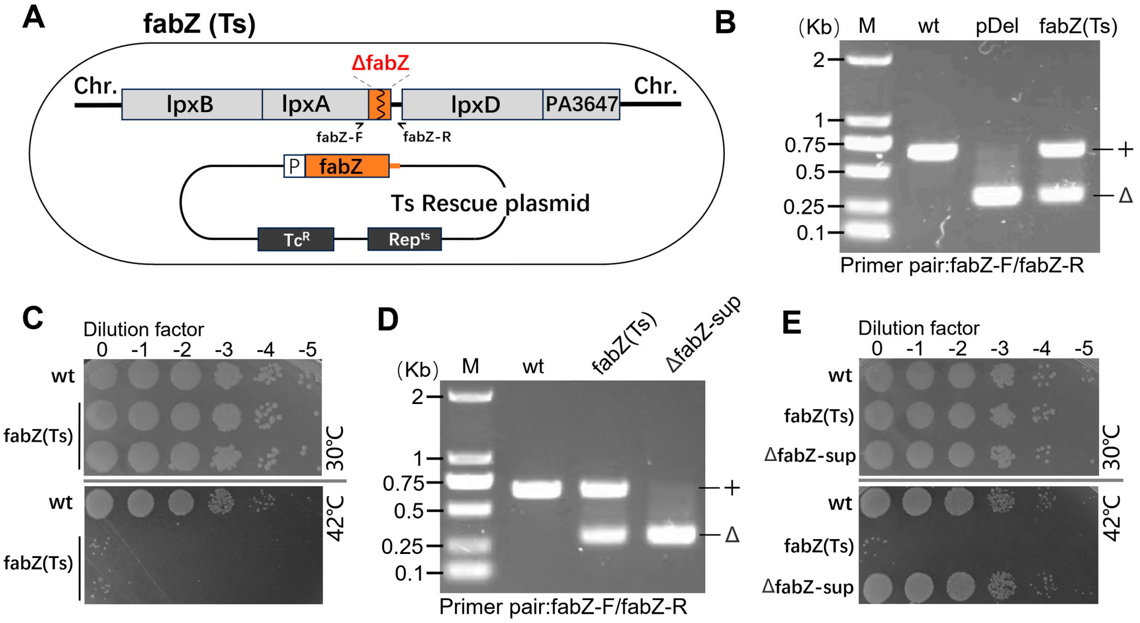

Supplementary Figure S1. Construction and characterization of the *fabZ* temperature-sensitive (ts) mutant *fabZ*(Ts) and suppressor mutant (Δ*fabZ*-sup) in *P. aeruginosa*. (A) Schematic representation of the *fabZ*(Ts) mutant construction. The chromosomal *fabZ* gene was deleted (Δ*fabZ*) and complemented with a temperature-sensitive (ts) rescue plasmid carrying the wild-type *fabZ* under its native promoter; Primer locations (*fabZ*-F and *fabZ*-R) for detecting wild-type and deletion alleles are indicated on the schematic; Tc^R: tetracycline resistance; Rep^{Ts}: temperature-sensitive replication origin. (B) PCR verification of *fabZ* deletion. The wild-type (wt) PAO1 strain shows an intact *fabZ* band (+), while the deletion plasmid control (pDel) and *fabZ*(Ts) mutant exhibit a smaller deletion fragment (Δ), confirming *fabZ* deletion on chromosome. (C) Spot-plating assay showing growth of wild-type and ts-mutant strains at 30°C (permissive temperature) and 42°C (restrictive temperature). The *fabZ*(Ts) strain failed to grow at 42°C, confirming its essentiality. (D) PCR confirmation of *fabZ* suppressor mutant (Δ*fabZ*-sup). The Δ*fabZ*-sup strain lacks the wild-type *fabZ* band (+) but retains the deletion band (Δ), verifying successful suppressor isolation. (E) Suppressor strain growth assay. While *fabZ*(Ts) was nonviable at 42°C, the Δ*fabZ*-sup strain successfully grew at both 30°C and 42°C, confirming suppression of the *fabZ*(Ts) lethal phenotype at 42°C.

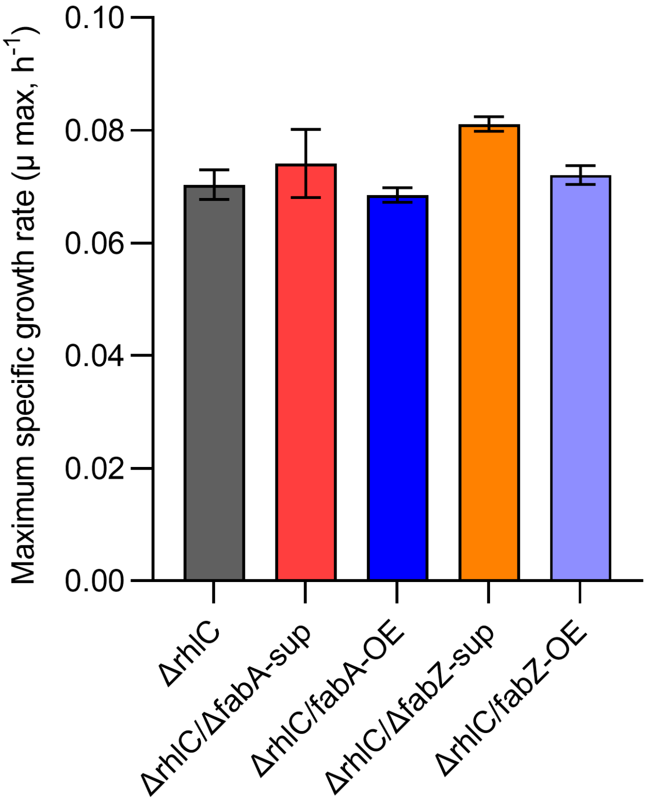

Supplementary Figure S2. Maximum specific growth rates (μ_{max}) of Δ*rhlC*-derived strains. Values were calculated from the exponential phase (12–48 h) using linear regression of $\ln_{(OD_{600})}$ vs. time. Data represent mean ± SD from three biological replicates.

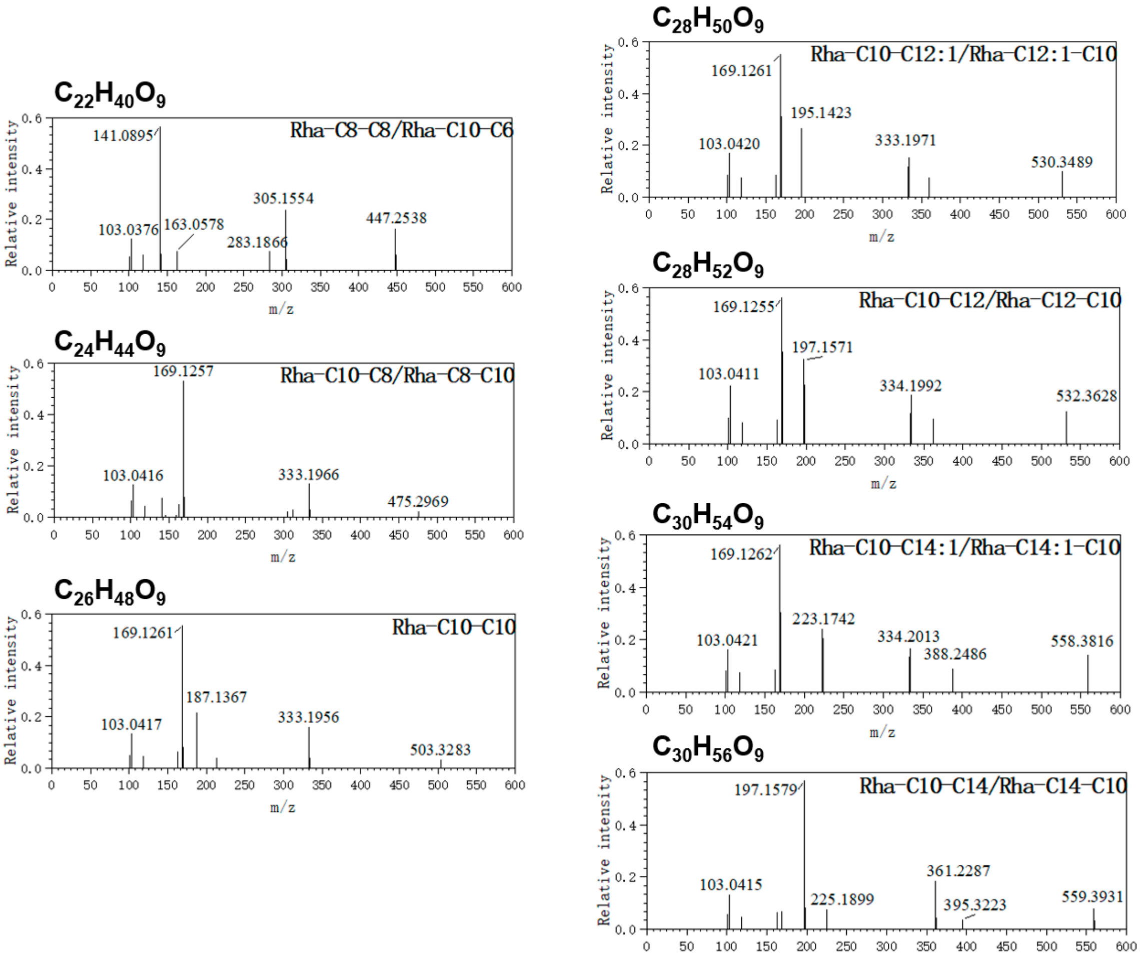

Supplementary Figure S3. MS2 spectra of various mRL congeners detected
