# Supplementary material for: Optimizing Rhamnolipid Performance by Modulating the Expression of Fatty Acid Synthesis Genes fabA and fabZ in Pseudomonas aeruginosa PAO1
Source: Genes (Basel). 2025 Apr 28;16(5):515. doi: 10.3390/genes16050515 (PMC12111694; doi:10.3390/genes16050515)
Supplement: Supplementary file 1 [file genes-16-00515-s001.zip › fabAZ_mRL_Supplementary Table S1.pdf]

Supplementary Table S1. Replicate abundance data for mRLs Composition in different strains

Strain	Structure	Replicate 1 (%)	Replicate 2 (%)	Replicate 3 (%)	Mean (%)	Std Dev
Δ*rhlC*	Rha-C8-C8 or Rha-C10-C6	4.51	3.1	3.5	3.7	0.73
Δ*rhlC*	Rha-C10-C8 or Rha-C8-C10	8.67	7.79	5.24	7.23	1.78
Δ*rhlC*	Rha-C10-C10	23.93	28.17	30.72	27.61	3.43
Δ*rhlC*	Rha-C10-C12:1 or Rha-C12:1-C10	26.98	29.66	34.28	30.31	3.69
Δ*rhlC*	Rha-C10-C12 or Rha-C12-C10	20.74	18.52	12.8	17.35	4.09
Δ*rhlC*	Rha-C10-C14:1 or Rha-14:1-C10	9.27	7.84	8.27	8.46	0.74
Δ*rhlC*	Rha-C10-C14 or Rha-C14-C10	5.9	4.93	5.19	5.34	0.51
Δ*rhlC*/Δ*fabA*-sup	Rha-C8-C8 or Rha-C10-C6	0.41	0.44	0.58	0.48	0.09
Δ*rhlC*/Δ*fabA*-sup	Rha-C10-C8 or Rha-C8-C10	3.45	2.79	3.78	3.34	0.51
Δ*rhlC*/Δ*fabA*-sup	Rha-C10-C10	15.03	13.57	10.22	12.94	2.46

Δ*rhlC*/Δ*fabA*-sup	Rha-C10-C12:1 or Rha-C12:1-C10	56.03	56.27	65.6	59.3	5.46
Δ*rhlC*/Δ*fabA*-sup	Rha-C10-C12 or Rha-C12-C10	5.78	7.39	6.27	6.48	0.82
Δ*rhlC*/Δ*fabA*-sup	Rha-C10-C14:1 or Rha-14:1-C10	16.03	15.42	10.76	14.07	2.88
Δ*rhlC*/Δ*fabA*-sup	Rha-C10-C14 or Rha-C14-C10	3.27	4.12	2.78	3.39	0.68
Δ*rhlC*/*fabA*-OE	Rha-C8-C8 or Rha-C10-C6	23.43	30.19	29.0	27.54	3.61
Δ*rhlC*/*fabA*-OE	Rha-C10-C8 or Rha-C8-C10	9.55	8.29	10.06	9.3	0.91
Δ*rhlC*/*fabA*-OE	Rha-C10-C10	11.78	9.17	9.35	10.1	1.45
Δ*rhlC*/*fabA*-OE	Rha-C10-C12:1 or Rha-C12:1-C10	23.75	19.52	14.96	19.41	4.39
Δ*rhlC*/*fabA*-OE	Rha-C10-C12 or Rha-C12-C10	7.67	6.78	6.85	7.1	0.5
Δ*rhlC*/*fabA*-OE	Rha-C10-C14:1 or Rha-14:1-C10	20.17	22.49	27.53	23.4	3.77

Δ*rhlC*/*fabA*-OE	Rha-C10-C14 or Rha-C14-C10	3.66	3.56	2.25	3.16	0.79
Δ*rhlC*/Δ*fabZ*-sup	Rha-C8-C8 or Rha-C10-C6	0.2	0.22	0.18	0.2	0.02
Δ*rhlC*/Δ*fabZ*-sup	Rha-C10-C8 or Rha-C8-C10	2.11	2.08	1.24	1.81	0.49
Δ*rhlC*/Δ*fabZ*-sup	Rha-C10-C10	42.83	40.93	46.56	43.44	2.87
Δ*rhlC*/Δ*fabZ*-sup	Rha-C10-C12:1 or Rha-C12:1-C10	47.38	48.28	41.8	45.82	3.51
Δ*rhlC*/Δ*fabZ*-sup	Rha-C10-C12 or Rha-C12-C10	1.71	1.61	1.54	1.62	0.08
Δ*rhlC*/Δ*fabZ*-sup	Rha-C10-C14:1 or Rha-14:1-C10	3.2	4.23	4.47	3.97	0.68
Δ*rhlC*/Δ*fabZ*-sup	Rha-C10-C14 or Rha-C14-C10	2.58	2.65	4.19	3.14	0.91
Δ*rhlC*/*fabZ*-OE	Rha-C8-C8 or Rha-C10-C6	20.07	17.7	23.11	20.29	2.71
Δ*rhlC*/*fabZ*-OE	Rha-C10-C8 or Rha-C8-C10	4.9	3.98	4.83	4.57	0.51
Δ*rhlC*/*fabZ*-OE	Rha-C10-C10	15.01	14.96	19.0	16.32	2.32

Δ*rhlC*/*fabZ*-OE	Rha-C10-C12:1 or Rha-C12:1-C10	29.83	34.59	28.3	30.9	3.28
Δ*rhlC*/*fabZ*-OE	Rha-C10-C12 or Rha-C12-C10	6.42	5.01	5.4	5.61	0.73
Δ*rhlC*/*fabZ*-OE	Rha-C10-C14:1 or Rha-14:1-C10	20.46	19.24	15.29	18.33	2.7
Δ*rhlC*/*fabZ*-OE	Rha-C10-C14 or Rha-C14-C10	3.32	4.51	4.08	3.97	0.6
